# Prognostic Significance of Lactate Dehydrogenase-to-Albumin Ratio and Neutrophil Percentage-to-Albumin Ratio in IgA Nephropathy

**DOI:** 10.3390/biomedicines14020318

**Published:** 2026-01-30

**Authors:** Balázs Sági, Tibor Vas, Sadra Salehi, Tibor József Kovács

**Affiliations:** 1 2nd Department of Internal Medicine and Nephrology, Diabetes Center, Clinical Center, Medical School, University of Pécs, 7624 Pécs, Hungary; 2 National Dialysis Center Pécs, 7624 Pécs, Hungary; 3 Faculty of Medicine, Medical School, University of Pécs, 7624 Pécs, Hungary

**Keywords:** lactate dehydrogenase-to-albumin ratio, neutrophil percentage-to-albumin ratio, chronic kidney disease, IgA nephropathy, renal and cardiovascular prognosis

## Abstract

**Background:** Inflammation plays a key role in the development of immunoglobulin A nephropathy (IgAN). The lactate dehydrogenase-to-albumin ratio (LAR) and neutrophil percentage-to-albumin ratio (NPAR) have emerged as markers reflecting inflammation and nutritional status. This study evaluated the prognostic significance of LAR and NPAR for predicting renal and cardiovascular (CV) outcomes in patients with IgAN. **Methods:** This study included 121 patients with biopsy-proven IgAN. The mean age was 43.6 ± 12.9 years, and 66% were male. Average follow-up time was 98.7 ± 63.3 months. The primary composite endpoints were total mortality, major CV events, and end-stage renal disease. Secondary endpoints, cardiovascular, or renal endpoints were also examined separately. Cox proportional hazards analyses were performed to evaluate these markers in predicting renal and CV prognosis. **Results:** Patients were divided into high and low groups for both LAR and NPAR based on ROC curve analysis. High LAR was linked to poorer outcomes for the primary composite endpoint (*p* = 0.03) and for separate renal (*p* = 0.018) and cardiovascular (*p* = 0.009) endpoints. Similarly, high NPAR was associated with worse primary (*p* = 0.02), renal (*p* = 0.039), and CV (*p* = 0.042) outcomes. In multivariate Cox regression analysis, high LAR remained an independent risk factor for the primary composite endpoint (HR = 4.165, 95% CI = 1.45–11.959, and *p* = 0.008) but not for renal or CV endpoints individually. **Conclusions:** High LAR and NPAR, as markers of inflammation and altered nutritional status, are associated with poorer renal and cardiovascular outcomes in IgAN and may serve as useful prognostic indicators.

## 1. Introduction

Immunoglobulin A nephropathy (IgAN) is the most common immunologically mediated glomerular disorder worldwide. A substantial proportion of cases progress to chronic kidney disease (CKD) and ultimately end-stage kidney disease (ESKD), with affected individuals frequently experiencing cardiovascular (CV) complications [1,2]. The pathogenesis of IgAN is characterized by a “multi-hit” autoimmune process in which aberrant, galactose-deficient IgA1 (Gd-IgA1) is produced. This abnormal IgA1 elicits the formation of autoantibodies, primarily IgG, resulting in immune complexes that deposit in the glomeruli, activate the complement, and induce inflammation, ultimately leading to glomerular injury, proteinuria, and fibrosis [1,2].

Lactate dehydrogenase (LDH), white blood cell count (WBC), and the serum albumin level are commonly accepted and used parameters in daily routine laboratory tests. However, the lactate dehydrogenase-to-albumin ratio (LAR) and the neutrophil percentage-to-albumin ratio (NPAR) are now being investigated as novel markers, as they are composite indices that integrate inflammatory activity with nutritional status. LDH is an intracellular enzyme that converts glucose to pyruvate under aerobic conditions and is then oxidized to acetyl-CoA, which enters the tricarboxylic acid (TCA) cycle in mitochondria to generate energy (ATP). The lactate signaling axis (LDH–lactate–lactylation) is now increasingly being demonstrated as playing an important physiological role in the development of diseases [3,4,5]. More and more drugs targeting lactate dehydrogenase and lactonization are being studied for disease treatment [6,7,8]. However, these studies are currently focused on the field of oncology. Under hypoxic conditions, the activation of LDH catalyzes the conversion of pyruvate to lactate during glycolysis [9]. When cell damage occurs, LDH could be released into the bloodstream from LDH-containing cells, leading to an increase in serum LDH levels. It has been shown to be an independent risk factor for poor outcomes in sepsis [10], acute pancreatitis [11], acute kidney disease [12], and tumors [13]. However, evidence regarding the predictive value of LDH for acute kidney injury (AKI) remains limited. Two studies found that elevated LDH was an independent risk factor for AKI after cardiac surgery [14,15].

Serum albumin is a commonly used laboratory parameter for assessing patients’ nutritional status and renal injury, especially in chronic disease [16,17]. Podocyte injury can disrupt the integrity of the glomerular filtration barrier, leading to proteinuria and hypoproteinemia [18]. Previous evidence suggests that decreased serum albumin levels are associated with higher mortality in sepsis and AKI cohorts [19,20]. In addition, a decline in the serum albumin level can serve as a predictor of mortality in advanced CKD patients [21].

Chronic inflammation and malnutrition significantly contribute to both overall and cardiovascular (CVD) mortality in patients with CKD [22]. The inflammatory markers are generally excluded from standard clinical laboratory tests, and their high cost further exacerbates the financial burden on the patients. Therefore, there is an obvious need to identify a convenient and cost-effective inflammatory–nutritional biomarker that shows an independent association with prognosis in patients with CKD stages G3a to G5. The neutrophil percentage-to-albumin ratio (NPAR) is an emerging biomarker for inflammatory nutrition and is calculated using the neutrophil percentage and albumin levels [23]. The NPAR provides a comprehensive reflection of the inflammatory and nutritional status of individuals. Recent studies have demonstrated that NPAR is a valuable prognostic biomarker for chronic and acute diseases, such as hypertension [24], cardiogenic shock, and myocardial infarction [25]. It is also associated with acute kidney injury in patients without chronic kidney disease undergoing percutaneous coronary intervention [26]. No studies have yet to elucidate the predictive role of NPAR in all-cause and CVD mortality among patients with CKD stages G3a to G5.

Higher NPAR scores generally indicate more severe inflammation and/or malnutrition state, as they reflect an increased percentage of neutrophils (a sign of inflammation) and/or decreased serum albumin levels (a sign of poor nutrition and reduced anti-inflammatory capacity). This novel index has shown prognostic utility across various conditions, including heart failure, diabetes, chronic kidney disease, and cancer, offering a more comprehensive assessment than individual markers, such as neutrophil count or albumin alone. NPAR can predict CV and all-cause mortality in patients with diabetes and CKD [27,28,29,30,31].

Several inflammatory and nutritional biomarkers have been investigated in CKD and IgAN, including C-reactive protein (CRP) [32], interleukins (e.g., IL-6) [33], tumor necrosis factor-alpha (TNF-α) [34], and composite indices such as the neutrophil-to-lymphocyte [35] or platelet-to-lymphocyte ratios [36]. Although these markers provide prognostic information, their routine clinical applicability is limited by cost, availability, or by reflecting only a single pathophysiological axis.

Serum albumin reflects the nutritional reserve and anti-inflammatory capacity, whereas the LDH and neutrophil percentage represent tissue injury, metabolic stress, and immune activation. Ratios that normalize inflammatory markers to albumin may therefore better capture the interaction between inflammation and nutritional status, a key determinant of disease progression in IgAN.

There is a lack of evidence in the literature for the renal and cardiovascular (CV) prognostic significance of LAR and NPAR in IgAN patients. In our study, we investigated a prognostic role of the LAR and NPAR in predicting renal and CV prognosis in IgAN patients.

## 2. Patients and Methods

### 2.1. Selection of Patients

A total of 150 patients with IgAN identified by renal biopsy at the University of Pécs Clinical Center’s 2nd Department of Internal Medicine, Nephrology, and Diabetes Centre between January 2003 and December 2018 were included in this single-center retrospective analysis. Just 2 of the 150 patients retracted their informed consent. Thirteen people receiving immunosuppressive treatment and fourteen people whose data was missing during follow-up were not included in the study. In the end, this study included 121 patients. For at least one to three months following the kidney biopsy, all patients underwent routine monitoring at the outpatient clinic. The follow-up period ranged from 4 to 300 months, with an average of 98.7 ± 63.3 months.

### 2.2. Clinical Data Collection

#### 2.2.1. Renal and Cardiovascular Endpoints

Acute coronary events (ACSs), stroke, cardiovascular (CV) outcomes (including overall mortality), and renal outcomes (ESKD development: renal replacement therapy was initiated or estimated glomerular filtration rate (eGFR) < 15 mL/min/1.73 m^2^) were included in the primary composite endpoint. The CV and renal outcomes were then examined independently as secondary endpoints.

#### 2.2.2. The Definition of Parameters

The LAR was measured as the absolute LDH level in a routine blood examination divided by serum albumin at the time of the renal biopsy. The NPAR is the neutrophil lymphocyte percentage divided by serum albumin at the time of the renal biopsy. In this study, cut-off values for the LAR and NPAR were determined using receiver operating characteristic (ROC) curve analysis. The optimal thresholds were selected based on the maximum Youden index (sensitivity + specificity − 1). For Kaplan–Meier survival analyses, patients were dichotomized into high and low groups according to these cut-off values. Routine laboratory examinations (hemoglobin, uric acid, total cholesterol, triglyceride, and high-density lipoprotein (HDL) cholesterol) and urine albumin measurements were also performed.

For the diagnosis of IgAN, every patient had a routine kidney biopsy, and the MEST-C classification from the histology was calculated. MEST-C is a histopathological scoring system that assesses the severity of the disease based on five biopsy findings: mesangial hypercellularity, endocapillary hypercellularity, segmental glomerulosclerosis, tubular atrophy/interstitial fibrosis, and crescents. Each component is scored individually, and the sum of these scores is used to predict renal prognosis and stratify risk for patients.

We used a validated ambulatory blood pressure monitoring system (ABPM) to measure the patients’ 24 h systolic and diastolic blood pressure oscillometrically (ABPM, Meditech, Hungary). Body mass index (BMI) was calculated by the standard method. The definition of metabolic syndrome (MetS) was stated by the National Cholesterol Education Program Adult Treatment Panel III (NCEP ATP III).

#### 2.2.3. Echocardiographic Measurement

Routine echocardiography was performed with an Aloka SSD 1400; two operators were involved in the study. Left ventricular mass (LVM) was calculated from 2D images of the left ventricular short-axis muscle area and apical left ventricular length (LVM = (5/6 area × length)). The assessment of the left ventricular mass index (LVMI g/m^2^) was obtained according to the formula by Devereux; the cardiac mass was also indexed with lean mass. LVMI was determined based on the Cornell criterion and indexed for height (in meters). The left ventricular ejection fraction (LVEF) was calculated by calculating the diastolic and systolic left ventricular volumes using the unidirectional Simpson method: EF = ((Dvol-Svol)/Dvol) × 100. Based on the measured LVMI and relative wall thickness (RWT) values, four different categories of the left ventricle geometry were identified: (1) normal (normal LVMI and normal RWT) (N), (2) concentric remodeling (normal LVMI and increased RWT) (CR), (3) eccentric hypertrophy (elevated LVMI and normal RWT) (EH), and (4) concentric hypertrophy (elevated LVMI and elevated RWT) (CH). Diastolic function was determined by mitral inflow and pulmonary venous flow based on conventional spectral Doppler measurements. We also measured the ratio of the E wave to the A wave (E/A ratio), the isovolumetric relaxation time (IVRT), and the deceleration time of the E wave. LVH was defined as abnormal RWT and/or LVMI.

### 2.3. Statistical Analysis

Continuous variables are often compared using t-tests and given as the mean ± SD. The Mann–Whitney U or Kruskal–Wallis tests were used to compare nonparametric variables, which are typically reported as medians with interquartile ranges. The χ2 test was utilized to compare categorical variables. Data were analyzed to determine the predictor variables or risk factors, univariate and multivariate linear regression analyses were employed.

Cox regression analysis was used to compare parameters and renal survival. Given the limited number of outcome events, the number of covariates included in multivariable Cox regression models was restricted. Only clinically relevant variables with significant associations in univariate analyses were entered into multivariable models. To avoid collinearity, LAR and NPAR were evaluated in separate regression analyses. Statistical significance was set at *p* < 0.05. Microsoft Excel 2021 (version 16) and SPSS 29.0.1.1 software were used.

## 3. Results

**Figure 1:**
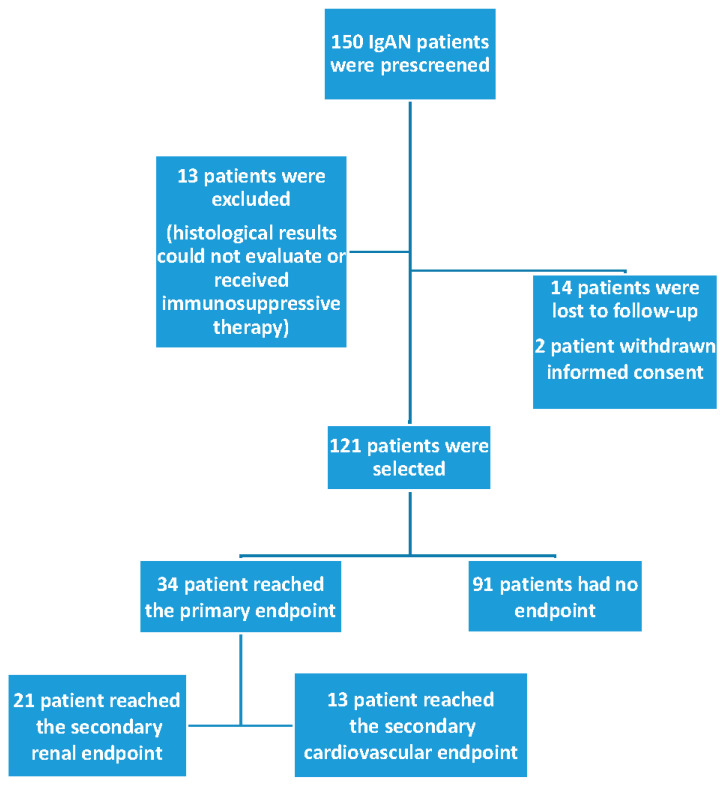
The flow chart of recruited patients.

The patient recruitment flowchart is presented in Figure 1. A total of 150 patients were prescreened, and 29 patients were excluded: 13 patients due to missing data or immunosuppressive therapy, 14 patients lost to follow-up, and 2 patients withdrew consent. In total, 121 patients with biopsy-proven IgAN were enrolled in this study. The average follow-up time was 98.7 ± 63.3 months (min.–max. 4–300 months). The mean age was 43.6 ± 12.9 years, and 66% were male. The baseline clinical data and laboratory results are shown in Table 1.

We divided the patients into two groups based on the ROC curve analysis of LAR and NPAR. There were significant differences in diastolic dysfunction occurrence, hemoglobin, and HDL cholesterol levels between the low- and high-LAR groups. There were also significant differences in age, 24 h systolic blood pressure, body mass index (BMI), left ventricular mass index (LVMI), and diastolic dysfunction between the low- and high-NPAR groups.

**Figure 2:**
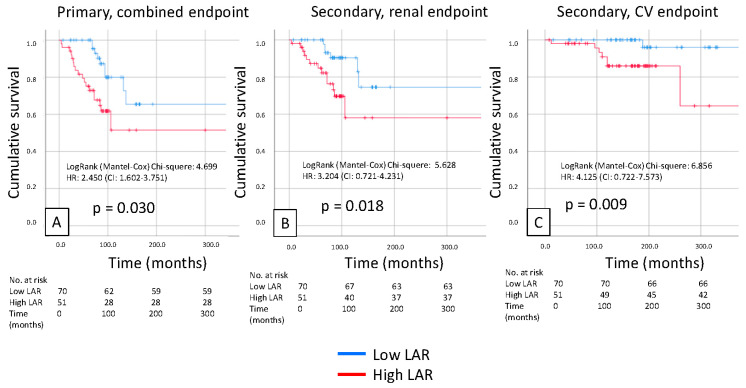
Kaplan-Meier curves show the primary combined (**A**), renal (**B**), and cardiovascular (**C**) endpoints for the high and low LAR IgAN patient groups.

**Figure 3:**
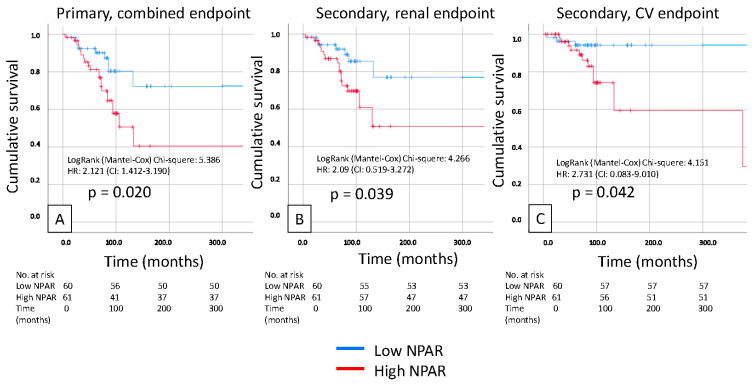
Kaplan-Meier curves show the primary combined (**A**), renal (**B**), and cardiovascular (**C**) endpoints for the high and low NPAR IgAN patient groups.

Kaplan–Meier analysis revealed that a high LAR was significantly associated with a poor primary composite endpoint (renal + CV; *p* = 0.03), and also, the secondary renal and CV endpoints separately predicted prognosis in patients with IgAN (*p* = 0.018 and *p* = 0.009). We also found similar results in the case of high NPAR, which was significantly associated with a poor primary composite endpoint (renal + CV; *p* = 0.02) and secondary endpoint prognosis in patients with IgAN (*p* = 0.039 and *p* = 0.042, respectively) (Figure 2 and Figure 3).

Pearson’s correlations showed significant correlations between LAR and age, hypertension, C-reactive protein (CRP), serum albumin, LDH, triglyceride (TG), HDL cholesterol, LVMI, and NPAR. We also found significant correlations between NPAR and age, hypertension, CRP level, serum albumin level, total cholesterol level, LVMI, and LAR (Table 2).

The primary composite endpoint occurred in 34 patients (28%), the secondary renal endpoint occurred in 21 patients (17%), and the cardiovascular endpoint occurred in 13 patients (11%). The primary and secondary endpoints in the LAR and NPAR groups are shown in Table 3.

The significant influencing factors of LAR were age, hypertension, serum albumin, HDL-cholesterol, LVMI, and CRP by multivariate regression analysis. The significant influencing factors of NPAR were age, eGFR, serum albumin level, LVMI, and LAR (Table 4).

The independent predictors of LAR were urine albuminuria and serum albumin, and the independent predictors of NPAR were urine albuminuria, serum albumin, and CRP by multivariate Cox regression analysis (Table 5).

The primary combined endpoint predictor was a high LAR but not NPAR in IgAN after adjusting for important clinicopathological parameters (HR = 1.844, 95% CI = 1.138–2.988, and *p* = 0.013) using multivariate Cox regression analysis (Table 6).

**Figure 4:**
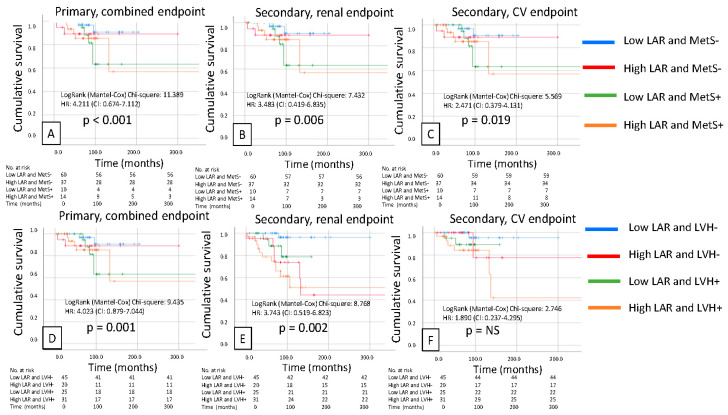
Kaplan–Meier curves show the primary composite (**A**), renal (**B**) and cardiovascular (**C**) endpoints for metabolic syndrome (MetS +/−) combined with LAR (high/low); and the primary composite (**D**), renal (**E**) and cardiovascular (**F**) endpoints for LAR (high/low) combined with left ventricular hypertrophy (LVH +/−).

**Figure 5:**
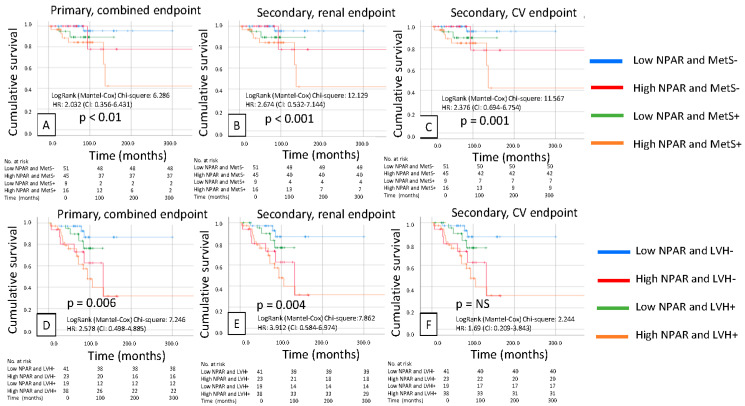
Kaplan–Meier curves show the primary composite (**A**), renal (**B**) and cardiovascular (**C**) endpoints for metabolic syndrome (MetS +/−) combined with NPAR (high/low); and the primary composite (**D**), renal (**E**) and cardiovascular (**F**) endpoints for NPAR (high/low) combined with left ventricular hypertrophy (LVH +/−).

Patients were then divided into four groups based on low and high LAR, presence or absence of metabolic syndrome (MetS), presence or absence of low or high LAR, and left ventricular hypertrophy (LVH). We found significantly worse survival in the primary (*p* < 0.001) and secondary renal (*p* = 0.006) and cardiovascular endpoints (*p* = 0.019) for the combination of high LAR and MetS, but for the combination of high LAR and LVH, except for the cardiovascular secondary endpoints, the primary (*p* = 0.001) and secondary renal endpoints (*p* = 0.002) were significant (Figure 4). We also created these groups for NPAR and MetS (primary endpoint, *p* < 0.001; secondary renal endpoint, *p* < 0.001; and secondary CV endpoint, *p* = 0.001) and LVH (primary endpoint, *p* = 0.006; secondary renal endpoint, *p* = 0.004; and secondary CV endpoint, *p* = NS), and the same results were obtained. The worst outcomes were observed for high NPAR combined with the presence of MetS and LVH (Figure 5).

## 4. Discussion

To our knowledge, this study provides novel evidence regarding the prognostic value of LAR and NPAR in IgAN. Our findings demonstrated that elevated values of both indices were strongly associated with adverse renal and cardiovascular (CV) outcomes. Our Kaplan–Meier analyses demonstrated a clear separation of risk according to LAR and NPAR categories.

### 4.1. LDH, Inflammation, and Renal Injury

Previous research on lactate dehydrogenase (LDH) supports the biological plausibility of our findings. An intracellular enzyme essential for lactate metabolism and glycolysis, LDH, is released into the bloodstream as a sign of hypoxia and tissue damage. In sepsis [10], acute pancreatitis [11], acute kidney injury (AKI) [12,14,15], and malignancies [13,17] elevated serum LDH, which has consistently been shown to be a poor prognostic marker. In population-based cohorts, it has also been linked to increased arterial stiffness and long-term CV risk [15]. Higher LDH levels have been associated with higher CV and all-cause mortality in patients receiving maintenance hemodialysis [16].

Serum albumin plays a dual role as a marker of nutritional reserve and as a negative acute-phase reactant with antioxidant and anti-inflammatory properties. In IgA nephropathy, hypoalbuminemia reflects not only protein loss due to glomerular injury but also systemic inflammation and reduced anti-inflammatory capacity. Therefore, normalizing inflammatory parameters to serum albumin allows for the integration of inflammatory burden and nutritional or anti-inflammatory reserve into a single composite index.

Normalization of lactate dehydrogenase or neutrophil to albumin reflects the balance between inflammation associated with tissue damage and the host’s ability to counterbalance the inflammation rather than the inflammation itself.

Chronic inflammation is a key factor in atherosclerosis and the progression of glomerular/kidney injury. The formation and deposition of aberrant IgA1 in the mesangium at the onset of IgAN involves several immunological steps. When inflammation occurs, cytokine activation occurs in the mesangium, a nonspecific process that leads to glomerular damage. Our observation that LAR predicts poor renal and cardiovascular outcomes in IgAN is consistent with these results and suggests that LDH normalized to albumin may reflect the combined burden of tissue damage, systemic inflammation, and malnutrition, as LDH alone is likely not sensitive enough to be of prognostic significance in chronic kidney disease.

LDH levels may contribute to disease progression in several ways. Experimental studies have shown that LDH activity and lactate metabolism play important roles in immune regulation and inflammatory signaling [5,6,7]. Given that IgAN is an immune-mediated glomerular disorder [1,2], LDH-related metabolic changes may exacerbate renal injury by modulating inflammatory pathways. Furthermore, the kidney is highly sensitive to hypoxia, and disturbances in lactate metabolism have been implicated in renal tissue injury and fibrosis [32,33,34,35]. These mechanisms provide a potential link between elevated LDH levels, renal progression, and CV complications. Importantly, neither serum albumin nor lactate dehydrogenase alone demonstrated comparable independent prognostic value in multivariable analyses, whereas LAR remained significantly associated with the primary composite endpoint after adjusting for relevant clinical covariates. This finding suggests that the ratio provides incremental prognostic information beyond its individual components.

### 4.2. Role of Albumin as a Counterbalance

As previously mentioned, the rationale for normalizing inflammatory markers to serum albumin is based on the dual function of albumin as an indicator of nutritional status and a negative acute-phase reactant. Hypoalbuminemia indicates protein loss, malnutrition, and reduced antioxidant and anti-inflammatory capacity, which are often observed in IgA nephropathy. Low serum albumin levels are associated with poor outcomes in sepsis [19,20], AKI [21], and chronic kidney disease [16,22]. In IgAN, podocyte injury and glomerular barrier disruption lead to proteinuria and hypoalbuminemia [18]. Albumin also exerts anti-inflammatory and antioxidant effects, and its reduction may exacerbate systemic inflammation and vascular dysfunction [22]. By combining LDH and albumin in a ratio, LAR may provide a more comprehensive index of the balance between tissue injury and nutritional reserve than either marker alone.

The biological relevance of lactate dehydrogenase is supported by experimental evidence demonstrating the segment-specific localization of LDH isoenzymes along the nephron, reflecting renal metabolic activity [37]. Beyond its metabolic role, lactate functions as an active signaling molecule via hydroxycarboxylic acid receptors and intercellular lactate shuttling, influencing inflammatory regulation, cellular communication, and vascular function [38,39,40]. These mechanisms provide a plausible biological basis for the association between LDH-related indices and adverse renal and cardiovascular outcomes.

### 4.3. NPAR as an Integrated Biomarker

Although LAR and NPAR are correlated due to the shared albumin denominator, they represent distinct biological processes. LAR primarily reflects tissue injury and metabolic stress, whereas NPAR reflects innate immune activation through neutrophil predominance. Consistent with this distinction, only LAR remained independently associated with the primary composite endpoint in multivariable analysis, indicating that the two indices are not fully redundant. NPAR has recently emerged as a prognostic biomarker in diverse clinical settings. Elevated NPAR levels have been shown to predict mortality in hypertension [24], cardiogenic shock, myocardial infarction [25], and contrast-induced AKI [26]. More recently, analyses of national health surveys have reported that higher NPAR values are associated with increased all-cause and CV mortality in chronic kidney disease [27,28,29,30,31]. These findings are consistent with our results, which demonstrate that NPAR independently predicts renal and CV outcomes in IgAN patients.

Mechanistically, NPAR integrates the inflammatory burden, as reflected by neutrophil predominance, with nutritional and anti-inflammatory reserves, represented by albumin. Neutrophil activation is a key driver of glomerular inflammation and progression in IgAN, while hypoalbuminemia reflects both protein loss and systemic catabolic stress. Therefore, NPAR may serve as a sensitive composite biomarker that reflects the two major pathophysiological axes of disease progression in IgAN, which are supported by our results.

### 4.4. Clinical Implications

Our findings have several important clinical implications. First, both the LAR and NPAR are inexpensive and routinely available laboratory parameters, making them practical tools for risk stratification in daily practice. Unlike more costly or specialized inflammatory markers, such as interleukin-6 or high-sensitivity CRP, these indices can be easily calculated from standard blood tests without an additional cost, but LAR or NPAR seems more sensitive for risk stratification. Examining the two parameters together more accurately indicates the body’s immunological activation than examining each factor (LDH, CRP) separately. Second, the ability of LAR and NPAR to predict both renal and CV outcomes underscores their value as integrated biomarkers in diseases where both pathways strongly influence long-term prognosis. Third, identifying high-risk patients based on LAR and NPAR may allow for the earlier initiation of renoprotective therapies, closer monitoring, and targeted CV risk reduction strategies.

Our findings highlighted the significance of this well-known and widely used marker in both IgAN and CKD by demonstrating a relationship between LAR, NPAR, and urinary albumin, as well as between the composite and renal outcomes and urinary albumin.

Regretfully, we were unable to identify any correlation between LAR and NPAR and the MEST-C score. This suggests that biological markers or their combination can more accurately predict the current disease activity than the crude classification of histological changes (0, 1, and 2 points) alone.

Our findings are consistent with those of Nigro et al., who demonstrated that readily available hematological parameters are closely associated with quantitative indices of renal structural damage. These observations support the concept that complex, integrated biomarkers—such as LAR and NPAR—may sensitively reflect disease activity and prognosis [41].

### 4.5. Relationship Between Renal Progression and Cardiovascular Mortality

The association between ESRD and CV mortality is well established. Patients with advanced CKD are at a 2- to 10-fold higher risk of CV events than non-CKD patients [29,30]. Almost half of all deaths in dialysis patients can be attributed to cardiovascular causes [29]. In this context, the combined, commonly used inflammatory markers studied showed a much higher sensitivity to cardiovascular and renal damage than either LDH or CRP alone. Our data support the ability of LAR and NPAR to predict renal and cardiovascular endpoints and highlight their potential role as markers of the severity of systemic disease and not just renal impairment. This dual prognostic relevance enhances their clinical utility and may justify their inclusion in IgAN risk prediction models. Chronic inflammation and endothelial damage sustained by IgAN or CKD can develop not only in the renal/glomerular capillaries but also in other predilection sites (e.g., the heart), which may also play a role in the development of pathological myocardial remodeling and atherosclerotic alterations.

Although several established inflammatory and nutritional biomarkers, such as interleukins or tumor necrosis factor-alpha, have prognostic value, these parameters are not routinely available in everyday nephrology practice. In contrast, LAR and NPAR are derived from inexpensive and universally accessible laboratory tests, supporting their potential utility as pragmatic prognostic markers.

LAR and NPAR are not intended to replace established IgA nephropathy risk prediction tools incorporating histological and clinical variables but are rather to complement them by capturing systemic inflammation and the nutritional status, which are not directly included in current prediction models.

### 4.6. Study Limitations and Future Perspectives

This study has several limitations. The cut-off values for LAR and NPAR were derived from this single-center cohort and may not be generalizable to other populations. Dynamic changes in these biomarkers over time were not assessed. The retrospective design and male predominance of the study population further limit generalizability and are also prone to residual confounding from comorbidities and medications that affect albumin levels and neutrophil counts. We did not examine the time change in LAR and NPAR; therefore, we cannot draw conclusions about the change in these two parameters. Finally, the predominance of male patients in our study may have limited its applicability to women. Future studies should aim to validate these findings in larger, multicenter, prospective cohorts with longer follow-up periods. In addition, mechanistic studies are needed to elucidate the biological pathways linking LAR and NPAR to disease progression in IgAN. The exploration of whether these biomarkers can guide therapeutic decision-making or whether dynamic changes in LAR and NPAR over time provide incremental prognostic value would also be of clinical relevance.

## 5. Conclusions

In conclusion, this study provides novel evidence that elevated LAR and NPAR levels, as special markers of inflammation within the human body, are strongly associated with adverse renal and cardiovascular outcomes in patients with IgAN. These composite indices reflect the interplay of inflammation, tissue injury, and nutritional status and may serve as practical and cost-effective tools for risk stratification in clinical practice. Validation in larger prospective studies is warranted, but our results suggest that LAR and NPAR may be promising integrated biomarkers to improve the prognostic evaluation and treatment of IgAN and also possibly immunologically active clinical disease.

## Figures and Tables

**Table 1:** Clinical data.

Parameter	Low LAR (n = 70)	High LAR (n = 51)	*p*	Low NPAR (n = 60)	High NPAR (n = 61)	*p*
Man/woman (n/%)	51/19 (73/27)	29/22 (57/43)	0.064	42/18 (70/30)	38/23 (62/38)	0.375
Age (year)	42.04 ± 12.66	46.61 ± 11.70	0.058	38.20 ± 12.65	48.59 ± 11.08	<0.001
Average 24 h systolic BP (mmHg)	122.9 (119.77–126.03)	124.60 (120.26–128.94)	0.539	121.44 (118.05–125.64)	125.86 (121.21–129.43)	0.042
Average 24 h diastolic BP (mmHg)	73.9 (71.77–76.03)	74.3 (71.52–77.08)	0.822	73.25 (71.3–75.76)	75.04 (72.87–78.97)	0.138
24 h pulse pressure (mmHg)	48.87 ± 6.84	50.42 ± 11.20	0.397	48.34 ± 8.35	50.77 ± 10.51	0.171
Metabolic parameters						
HT (n, %)	37 (68.5)	42 (82.4)	0.103	41 (68.3)	49 (80.3)	0.133
BMI (kg/m^2^)	26.00 ± 4.06	27.13 ± 4.93	0.200	25.67 ± 3.99	27.50 ± 5.04	0.029
Dyslipidemia (n, %)	23 (42.6)	26 (51)	0.394	24 (40.0)	31 (50.8)	0.236
DM (n, %)	9 (16.7)	15 (29.4)	0.122	11 (18.3)	17 (27.9)	0.217
eGFR (mL/min/1.73 m^2^)	74.37 ± 10.05	74.50 ± 10.54	0.951	72.55 ± 8.61	74.58 ± 11.52	0.287
Duration of IgAN (year)	10.02 ± 9.63	9.43 ± 9.63	0.755	8.00 ± 7.37	11.34 ± 11.46	0.059
Smoking (n, %)	9 (16.7)	9 (17.6)	0.895	8 (13.3)	12 (19.7)	0.352
MetS (n, %)	11 (15.7)	14 (27.5)	0.114	9 (15.0	16 (26.2)	0.129
Albumin-related parameters						
LAR	7.17 ± 1.04	10.03 ± 1.53	0.001	7.76 ± 1.44	9.30 ± 2.09	<0.001
NPAR	1.50 ± 0.26	1.61 ± 0.25	0.023	1.31 ± 0.15	1.77 ± 0.16	<0.001
Echocardiographic parameters						
LVEF (%)	62.8 ± 6.25	62.3 ± 6.60	0.729	61.7 ± 6.29	63.8 ± 6.43	0.088
LVMI (g/m^2^)	103.64 ± 24.05	109.76 ± 22.03	0.182	102.44 ± 20.74	112.50 ± 25.01	0.020
LVEDD (cm)	5.68 ± 5.63	5.98 ± 6.51	0.803	4.96 ± 0.44	6.47 ± 7.88	0.147
DD (n/%)	16 (25.9)	31 (60.8)	0.001	19 (31.7)	38 (62.2)	0.001
Laboratory results						
Hb (g/dL)	14.22 (13.77–14.67)	13.40 (12.97–13.83)	0.010	13.95 (13.04–14.87)	13.51 (12.99–14.21)	0.076
AU (mg/24 h)	413.61 ± 607.47	526.13 ± 691.69	0.377	403.85 ± 608.1	497.85 ± 643.8	0.207
UA (umol/L)	323.19 (297.21–349.17)	319.75 (295.57–343.93)	0.847	324.11 (297.43–350.78)	338.80 (308.78–369.13)	0.211
Total cholesterol (mmol/L)	4.83 (4.56–5.10)	5.00 (4.65–5.35)	0.456	5.05 (4.76–5.45)	4.88 (4.67–5.17)	0.215
HDL cholesterol (mmol/L)	1.21 ± 0.32	1.45 ± 0.56	0.008	1.29 ± 0.33	1.27 ± 0.56	0.381
TG (mmol/L)	1.82 ± 1.30	1.69 ± 1.00	0.547	1.69 ± 1.05	1.78 ± 1.13	0.339
TG/HDL	1.75 ± 1.56	1.46 ± 1.04	0.281	1.46 ± 1.15	1.70 ± 1.37	0.154
LDH (U/L)	306.42 ± 45.31	397.0 ± 63.56	0.001	337.29 ± 60.54	362.39 ± 79.27	0.04
CRP (mg/L)	5.13 ± 10.41	7.44 ± 17.59	0.367	4.09 ± 12.88	8.18 ± 15.20	0.272
se Albumin (g/L)	42.86 ± 3.87	39.71 ± 3.70	0.001	43.62 ± 3.78	41.51 ± 3.30	0.001
Pathological lesions						
M (n/%)	27 (38)	22 (43)	0.627	24 (41)	24 (39)	0.649
E (n/%)	0 (0)	1 (2)	0.335	1 (2)	0 (0)	0.287
S (n/%)	10 (14)	10 (19)	0.919	9 (15)	13 (2)	0.547
T (n/%)	26 (37)	30 (59)	0.471	18 (30)	35 (56)	0.054
C (n/%)	14 (20)	14 (27)	0.741	10 (17)	16 (26)	0.310

BP: blood pressure; HT: hypertension; DM: diabetes mellitus; BMI: body mass index; eGFR: estimated glomerular filtration rate; IgAN: immunoglobulin A nephropathy; MetS: metabolic syndrome; LAR: lactate dehydrogenase-to-albumin ratio; NPAR: neutrophil percentage-to-albumin ratio; LVEF: left ventricular ejection fraction; LVMI: left ventricular mass index; LVEDD: left ventricular end-diastolic diameter; DD: diastolic dysfunction; AU: urine albuminuria; UA: uric acid; HDL: high-density lipoprotein; TG: triglyceride; se: serum; CRP: C-reactive protein; M: mesangial hypercellularity; E: endocapillary hypercellularity; S: segmental glomerulosclerosis; T: tubular atrophy/interstitial fibrosis; and C: cellular crescents.

**Table 2:** Pearson’s correlations.

	LAR		NPAR	
	r	*p*	r	*p*
Age	0.232	0.017	0.368	<0.001
HT	0.215	0.027	0.269	0.007
CRP	0.897	<0.001	0.411	<0.001
se Albumin	−0.411	<0.001	−0.620	<0.001
LDH	0.393	<0.001	0.930	NS
TG	0.320	0.001	0.110	NS
HDL cholesterol	0.318	<0.001	0.034	NS
Total cholesterol	0.153	NS	0.197	0.036
LVMI	0.235	0.014	0.325	0.001
LAR	-	-	0.354	0.001
NPAR	0.471	<0.001	-	-

LAR: lactate dehydrogenase-to-albumin ratio; NPAR: neutrophil percentage-to-albumin ratio; HT: hypertension; CRP: C-reactive protein; se: serum; LDH: lactate dehydrogenase; LVMI: left ventricular mass index; HDL: high-density lipoprotein; and TG: triglyceride; NS: not significant.

**Table 3:** Primary and secondary renal and CV endpoints in different LAR and NPAR groups.

IgAN Patients (n = 121)	Low LAR (n = 70)	High LAR (n = 51)	Low NPAR (n = 60)	High NPAR (n = 61)
Renal endpoints (n/%)	7 (10)	14 (27)	7 (12)	14 (23)
CV endpoints (n/%)	4 (6)	9 (17)	3 (5)	10 (16)
Primary combined endpoints (n/%)	11 (16)	23 (45)	10 (17)	24 (39)

IgAN: immunoglobulin A nephropathy; LAR: lactate dehydrogenase-to-albumin ratio; NPAR: neutrophil percentage-to-albumin ratio; and CV: cardiovascular.

**Table 4:** Multivariate regression analysis of LAR and NPAR influencing factors.

LAR	MULTIVARIATE ANALYSIS			
Parameters	B	95% CI for B Lower	95% CI for B Upper	*p*
Gender	−0.468	−1.254	0.318	0.241
Age	0.036	0.006	0.066	0.017
Dyslipidemia	−0.256	−1.008	0.495	0.500
Obesity	0.812	−0.056	1.680	0.066
HT	0.960	0.110	1.810	0.027
DM	0.630	−0.257	1.516	0.162
MetS	0.738	−0.174	1.650	0.111
24BPsyst (mmHg)	0.010	−0.018	0.038	0.484
24BPdiast (mmHg)	0.020	−0.023	0.063	0.364
eGFR (ml/min)	−0.009	−0.019	0.001	0.086
Hb (g/dL)	−0.190	−0.420	0.040	0.104
AU (mg/day)	0.001	0.001	0.001	0.185
se Albumin (g/L)	−0.204	−0.285	−0.122	<0.001
Total cholesterol (mmol/L)	−0.018	−0.354	0.318	0.915
HDL cholesterol (mmol/L)	1.295	0.545	2.045	<0.001
TG (mmol/L)	−0.282	−0.604	0.040	0.085
LVMI (g/m^2^)	0.018	0.002	0.034	0.030
CRP (mg/L)	0.032	0.006	0.058	0.015
NPAR	MULTIVARIATE ANALYSIS			
Parameters	B	95% CI for B lower	95% CI for B upper	*p*
Gender	−0.015	−0.123	0.093	0.785
Age	0.008	0.005	0.012	<0.001
Dyslipidemia	0.028	−0.075	0.130	0.597
Obesity	0.064	−0.054	0.182	0.282
HT	0.089	−0.027	0.205	0.132
DM	0.026	−0.093	0.146	0.663
MetS	0.040	−0.084	0.165	0.522
24BPsyst (mmHg)	0.002	−0.002	0.006	0.246
24BPdiast (mmHg)	0.002	−0.004	0.008	0.485
eGFR (ml/min)	−0.002	−0.003	0.001	0.008
Hb (g/dL)	−0.023	−0.054	0.007	0.136
AU (mg/day)	1.801	0.001	0.001	0.658
se Albumin (g/L)	−0.038	−0.049	−0.028	<0.001
Total cholesterol (mmol/L)	−0.014	−0.058	0.030	0.537
HDL cholesterol (mmol/L)	−0.034	−0.142	0.074	0.534
TG (mmol/L)	0.013	−0.034	0.059	0.590
LVMI (g/m^2^)	0.003	0.001	0.005	0.003
LDH (U/L)	0.001	0.001	0.001	0.340
CRP (mg/L)	0.003	−0.001	0.007	0.125
LAR	0.051	0.025	0.077	<0.001

LAR: lactate dehydrogenase-to-albumin ratio; NPAR: neutrophil percentage-to-albumin ratio; HT: hypertension; DM: diabetes mellitus; MetS: metabolic syndrome; BP: blood pressure; eGFR: estimated glomerular filtration rate; Hb: hemoglobin; AU: urine albuminuria; se: serum; HDL: high-density lipoprotein; TG: triglyceride; LVMI: left ventricular mass index; LDH: lactate dehydrogenase; and CRP: C-reactive protein.

**Table 5:** Multivariate Cox regression analysis of LAR and NPAR influencing factors.

LAR	Exp(B)	95.0% CI for Exp(B)	95.0% CI for Exp(B)	*p*
Gender	1.584	0.719	3.489	0.253
Age	1.011	0.979	1.044	0.511
Dyslipidemia	1.135	0.471	2.734	0.777
Obesity	0.589	0.237	1.464	0.255
HT	0.678	0.251	1.831	0.443
DM	1.640	0.170	15.833	0.669
MetS	0.773	0.079	7.567	0.825
BP systolic	0.991	0.959	1.023	0.562
BP diastolic	0.992	0.937	1.049	0.777
IgAN duration	0.974	0.933	1.016	0.215
eGFR	0.995	0.983	1.006	0.368
AU	1.001	1.000	1.001	0.002
se Alb	0.863	0.775	0.962	0.008
Total cholesterol	1.138	0.773	1.676	0.513
HDL cholesterol	1.577	0.705	3.523	0.267
TG	0.875	0.558	1.373	0.561
LVMI	1.001	0.982	1.020	0.952
CRP	1.012	0.988	1.037	0.314
NPAR	Exp(B)	95.0% CI for Exp(B)	95.0% CI for Exp(B)	*p*
Gender	0.872	0.412	1.845	0.720
Age	1.014	0.979	1.050	0.433
Dyslipidemia	0.609	0.263	1.413	0.248
Obesity	1.003	0.414	2.426	0.996
HT	2.534	0.790	8.129	0.118
DM	1.040	0.110	9.828	0.973
MetS	1.032	0.110	9.676	0.978
IgAN duration	1.000	0.966	1.036	1.000
eGFR	0.995	0.984	1.007	0.394
AU	1.001	1.000	1.002	0.001
se Albumin	0.828	0.745	0.920	0.001
Total cholesterol	0.831	0.567	1.218	0.343
HDL cholesterol	1.093	0.477	2.506	0.833
TG	1.134	0.777	1.656	0.515
LVMI	0.991	0.972	1.010	0.351
CRP	1.032	1.009	1.056	0.007

LAR: lactate dehydrogenase-to-albumin ratio; NPAR: neutrophil percentage-to-albumin ratio; HT: hypertension; DM: diabetes mellitus; MetS: metabolic syndrome; IgAN: immunoglobulin A nephropathy; BP: blood pressure; eGFR: estimated glomerular filtration rate; AU: urine albuminuria; se: serum; HDL: high-density lipoprotein; TG: triglyceride; LVMI: left ventricular mass index; LDH: lactate dehydrogenase; and CRP: C-reactive protein.

**Table 6:** Cox regression analysis of the primary and secondary renal and CV endpoints affecting parameters.

Primary Endpoint				
	Exp(B)	95.0% CI for Exp(B)	95.0% CI for Exp(B)	*p*
Gender	0.432	0.107	1.751	0.240
Age	1.033	0.986	1.082	0.168
Dyslipidemia	6.469	1.799	23.261	0.004
Obesity	1.285	0.248	6.650	0.765
HT	0.110	0.012	0.972	0.047
DM	10,102.717	0.001	5.395	0.915
MetS	0.001	0.001	2.015	0.906
BP systolic	0.957	0.899	1.020	0.179
BP diastolic	1.141	1.019	1.277	0.022
IgAN duration	1.021	0.966	1.080	0.458
eGFR	0.984	0.965	1.002	0.086
AU	1.001	1.000	1.002	0.003
se Alb	1.041	0.891	1.217	0.610
Total cholesterol	0.836	0.499	1.399	0.495
HDL cholesterol	1.471	0.377	5.745	0.579
TG	1.498	0.990	2.266	0.056
LVMI	1.012	0.986	1.039	0.358
CRP	1.013	0.976	1.051	0.499
LAR	4.165	1.450	11.959	0.008
NPAR	7.213	0.438	118.860	0.167
Secondary renal endpoint				
	Exp(B)	95.0% CI for Exp(B)	95.0% CI for Exp(B)	*p*
Gender	2.060	0.411	10.330	0.380
Age	1.043	0.984	1.105	0.160
Dyslipidemia	4.888	0.849	28.144	0.076
Obesity	0.716	0.094	5.461	0.748
HT	0.161	0.013	1.977	0.153
DM	30,383.446	0.001	2.586	0.915
MetS	0.001	0.001	4.545	0.919
BP systolic	0.978	0.926	1.034	0.436
BP diastolic	1.055	0.950	1.172	0.314
IgAN duration	0.996	0.921	1.077	0.916
eGFR	0.971	0.946	0.996	0.024
AU	1.002	1.001	1.003	<0.001
se Albumin	1.027	0.833	1.266	0.804
Total cholesterol	0.487	0.219	1.087	0.079
HDL cholesterol	6.722	0.951	47.524	0.056
TG	1.943	1.106	3.413	0.021
LVMI	1.036	0.996	1.077	0.079
CRP	1.015	0.970	1.061	0.521
LAR	3.204	0.721	14.231	0.126
NPAR	20.389	0.519	800.272	0.107
Secondary CV endpoint				
	Exp(B)	95.0% CI for Exp(B)	95.0% CI for Exp(B)	*p*
Gender	0.116	0.005	2.471	0.168
Age	1.126	0.984	1.288	0.085
Dyslipidemia	12.623	0.907	175.585	0.059
Obesity	26.225	1.334	515.673	0.032
HT	0.003	0.001	141,947.595	0.517
DM	1190.626	0.001	1.506	0.952
MetS	0.001	0.001	1.301	0.922
BP systolic	0.876	0.743	1.034	0.117
BP diastolic	1.432	1.067	1.921	0.017
IgAN duration	1.068	0.962	1.185	0.217
eGFR	0.985	0.952	1.019	0.377
AU	1.001	0.999	1.002	0.461
Alb	1.231	0.938	1.614	0.133
Total cholesterol	1.007	0.454	2.233	0.986
HDL cholesterol	0.631	0.016	25.354	0.807
TG	1.542	0.657	3.618	0.320
LVMI	1.024	0.977	1.073	0.315
CRP	0.994	0.915	1.080	0.884
LAR	4.125	0.722	23.573	0.111
NPAR	8.731	0.083	919.010	0.362

HT: hypertension; DM: diabetes mellitus; MetS: metabolic syndrome; IgAN: immunoglobulin A nephropathy; BP: blood pressure; eGFR: estimated glomerular filtration rate; AU: urine albuminuria; se: serum; HDL: high-density lipoprotein; TG: triglyceride; LAR: lactate dehydrogenase-to-albumin ratio; NPAR: neutrophil percentage-to-albumin ratio; LVMI: left ventricular mass index; and CRP: C-reactive protein.

## Data Availability

The data underlying this article cannot be shared publicly because of Hungarian regulations and the privacy of individuals who participated in the study. The data could be shared upon reasonable request to the corresponding author if accepted by the Regional Committee for Medical and Health Research Ethics and local Data Protection Officials.
